# Protective role of heme oxygenase-1 in fatty liver ischemia–reperfusion injury

**DOI:** 10.1007/s00795-018-0205-z

**Published:** 2018-08-31

**Authors:** Shaowei Li, Masayuki Fujino, Terumi Takahara, Xiao-Kang Li

**Affiliations:** 1 Division of Transplantation Immunology, National Research Institute for Child Health and Development, 2-10-1 Okura, Setagaya-ku, Tokyo, 157-8535 Japan; 2 AIDS Research Center, National Institute of Infectious Diseases, Tokyo, Japan; 3 Third Department of Internal Medicine, University of Toyama, Toyama, Japan

**Keywords:** Fatty liver, HO-1, Ischemia reperfusion

## Abstract

Ischemia–reperfusion (IR) injury is a kind of injury resulting from the restoration of the blood supply after blood vessel closure during liver transplantation and is the main cause of graft failure. The pathophysiological mechanisms of hepatic IR include a variety of oxidative stress responses. Hepatic IR is characterized by ischemia and hypoxia inducing oxidative stress, immune response and apoptosis. Fat-denatured livers are also used as donors due to the lack of liver donors. Fatty liver is less tolerant to IR than normal liver. Heme oxygenase (HO) is an enzyme that breaks down hemoglobin to bilirubin, ferrous iron and carbon monoxide (CO). Inducible HO subtype HO-1 is an important protective molecule in mammalian cells used to improve acute and chronic liver injury owing to its characteristic anti-inflammatory and anti-apoptotic qualities. HO-1 degrades heme, and its reaction product CO has been shown to reduce hepatic IR injury and increase the survival rate of grafts. As an induced form of HO, HO-1 also exerts a protective effect against liver IR injury and may be useful as a new strategy of ameliorating this kind of damage. This review summarizes the protective effects of HO-1 in liver IR injury, especially in fatty liver.

## Introduction

Ischemia is a kind of restriction or interruption of the blood supply to tissues that leads to ischemic damage in the tissue due to a vigorous metabolism. The blood that circulates after ischemia triggers a series of events which may exacerbate the original damage. This phenomenon is called reperfusion damage [1, 2].

Many cells in the liver are vulnerable to IR damage. The occurrence of hepatic IR damage is correlated with a variety of factors [3, 4]. The pathophysiological response to this injury is primarily related to microcirculatory interference induced by reactive oxygen species (ROS) [5]. The activation of ischemia and oxidative stress triggers a series of protein kinases aggregated on transcription factors to regulate the expression of inflammatory factors. These inflammatory factors include chemokines (e.g., chemokines keratinocyte chemoattractant [CXCL1], macrophage inflammatory protein-2 [MIP-2 or CXCL2] and the receptors expressed on the surface of cells, such as chemokine [C-X-C motif] receptor 1&2 [CXCR1&2]), inflammatory cytokines (e.g., interleukin-23 [IL-23], interleukin-12 [IL-12] and tumor necrosis factor α [TNF-α]), transcription factors (e.g., nuclear factor kappa B [NF-κB]) and adhesion molecules (e.g., intracellular adhesion molecule [ICAM-1], vascular cell adhesion protein 1 [VCAM-1] and P-selectin) [6–10]. The resulting local inflammation is further aggravated by the recruitment of leukocytes, especially neutrophils, which may be the cornerstone of liver ischemia and reperfusion damage [11, 12].

In addition, IR injury causes programmed death of endothelial cells, and a large amount of apoptosis of vascular endothelial cells can lead to thrombosis in liver [13]. The series of lesions observed during liver IR injury can trigger inflammatory reactions of other tissues in the body, primarily in the lungs [14].

Hepatic IR injury is a complex and multi-factorial pathophysiological process. The post-ischemic liver is severely damaged and stressed, thus creating a biological environment different from hepatectomy. Although many of the mechanisms of liver regeneration that occur after hepatectomy also play a role after IR injury, in contrast to liver resection, the hepatocytes remaining after IR are subjected to high levels of stress injury, and inflammation affects hepatocyte proliferation [15].

During liver repair after IR injury, the interactions of non-parenchymal cells, such as Kupffer cells and stellate cells, are intimately involved in hepatic remodeling [16, 17]. The proliferation of hepatocytes after IR begins in the perivascular area and is associated with the expression of CXCR2 on myeloid cells instead of hepatocytes [7]. The proliferation and differentiation of hepatocytes is governed by CXCR2 after IR injury. Antagonist of CXCR4 was found to improve the hepatic recovery after IR injury, suggesting that CXCR4 is detrimental to liver regeneration [18].

HO, a universal and essential enzyme, is observed in almost all eukaryotes [19, 20]. The HO enzyme system is the rate-limiting step dominating the conversion of heme to biliverdin, CO and the ferrous form Fe^2+^ [21]. HO-1 is one of the three different HO subtypes: HO-1, HO-2 and HO-3 [22, 23]. HO-2 and HO-3 are produced constitutively as the heme-binding enzymes in normal cells, while HO-1 is an integral type I transmembrane protein of smooth endoplasmic reticulum [24] and an induced form of the enzyme. HO-2 is the primary subtype of HO in the brain and testis under common physiological conditions [25]. In contrast, the expression of HO-1 is relatively low, except for in the spleen, which has a high constitutive expression. HO-1 overexpression is a critical cell-protective mechanism for activation in tissues and organs under stressful conditions, such as inflammation and apoptosis [26, 27], ischemia [28] and hypoxia [29]. HO-1 is also involved in maintaining the balance of antioxidants and oxidants in the process of cell damage [28, 30]. Recent studies have renewed our understanding of the signaling pathway of HO as a coordinated protective system [31].

The HO-1 expression may be a most promising therapeutic target for relieving IR injury. This article reviews the status of research regarding the cell-protective function of HO-1 in hepatic IR injury. Furthermore, we will explore the possible therapeutic effects of HO-1 in hepatic IR injury.

## Underlying mechanism and approaches to treating hepatic IR injury in patients with fatty liver

Over the past 20 years, the number of patients undergoing liver transplantation has been steadily increasing due to the rising incidence of cirrhosis [32, 33]. At present, non-alcoholic fatty liver disease (NAFLD)-induced end-stage liver disease is the most common indication for liver transplantation [32]. The high incidence of NAFLD increases the demand for liver donor organs, but many fatty livers in donor pools are considered unsuitable as transplantation donors. A recent study found that 78% of potential liver donors had varying degrees of fatty liver and came from donors with a body mass index (BMI) exceeding 28 [34]. The results of another study showed that 21% of cadaveric donors had moderate fatty degeneration in the liver [35]. A retrospective study found that 40% of grafts could not be used and had to be discarded due to varying degrees of steatosis [36].

Because of the extreme imbalance in supply and demand, marginal liver donor use has increased, with grafts now considered from older donors, donations after heart death and (most commonly) donors with mild or moderate fatty liver [37]. Fatty liver is considered an edge donor for liver transplantation. Increased organ damage can increase the susceptibility to IR damage, which can subsequently lead to low rates of transplantation and patient survival. In addition, the rates of initial dysfunction and primary non-function have also increased.

Generally speaking, fatty livers are particularly susceptible to IR damage. Animal experimental results have shown that the IR damage mechanism differs between normal and fatty liver [38]. NFκB subunit p65 activation plays a key role in steatotic liver transplantation-induced IR injury [39]. The main form of hepatic parenchymal cell death is apoptosis in ischemic and non-steatosis liver; in contrast, fatty liver develops necrosis after IR injury [40]. Macrovesicular steatosis liver has a lower tolerance than microvesicular steatosis liver [41]. Fatty liver is generally defined as liver with degeneration of vesicular lipid droplets. Data from clinical studies support the correlation between the extent of hepatic steatosis and increased vulnerability to IR damage [42, 43]. Due to the presence of lipid droplets that cause nuclear translocation, the prognosis of the recovery of liver function is usually poor and it is more harmful to the liver after IR than liver function recovery. However, the fatty degeneration of microbubbles is defined as the presence of compound lipid droplets of microvesicles without the generation of nuclear translocation, and the damage to the liver is considered to be smaller than macrovesicular lipid droplets existence in liver after the IR process.

## Roles of non-parenchymal and parenchymal cells in IR injury of the liver

### Kupffer cell activation

Kupffer cells are liver-resident macrophages located in the sinusoidal lumen, mainly at sinusoidal branch points. They constitute liver sinusoidal cells along with other kinds of cells, such as endothelial cells, stellate cells and dendritic cells. All of these non-parenchymal cells interact in the process of hepatic IR damage. Previously, it was thought that Kupffer cells were immobilized or ‘fixed’. Now, studies have shown that they move along the sinusoid to the damaged areas of the liver [44]. Kupffer cells contribute to liver damage during ischemia and reperfusion. During the early stage of reperfusion, Kupffer cells alter their morphology and produce large amounts of ROS induced by ischemia followed by reperfusion [45]. In addition, hepatocyte apoptosis occurs in the phases of IR, resulting in the release of endogenous damage-associated molecular patterns (DAMPs), such as high mobility group box-1 (HMGB1) and denatured nuclear DNA. DAMPs activate Kupffer cells by binding to Toll-like receptor (TLR), which in turn generates an inflammatory reaction, producing a great deal of pro-inflammatory cytokines, such as TNF-α and interleukin-1 (IL-1) [46]. These pro-inflammatory cytokines play an essential role in aggravated hepatic IR damage. The release of cytokines through oxygen-free radicals and activated Kupffer cells facilitates the removal of protein–polysaccharide complexes from the surface of vascular endothelial cells and increases the exposure of adhesion molecules on the surface of endothelial cells. This phenomenon promotes the adhesion of neutrophils and platelets to the sinus endothelial cells, thereby exacerbating endothelial cell injury and ultimately causing serious damage to microcirculation and aggravating the degree of tissue ischemia.

Kupffer cells are considered to play an important role in liver IR damage. The initial stages of reperfusion make dramatic morphological changes to activated Kupffer cells and compel them to extend into the central sinusoid [47]. Activated Kupffer cells are major contributors to the release of not only large amounts of cytokines but also intracellular ROS [48]. Evidence suggests that liver parenchymal cell damage occurs in the hypoxic phase of IR injury. Pharmacological preconditioning for protection against hepatic IR injury by reducing Kupffer cell activation has been reported. Indeed, Mosher et al. [49] reported that gadolinium chloride relieves liver cell injury caused by liver IR by inhibiting Kupffer cell activity. Kupffer cells are a kind of macrophage located in the hepatic sinusoid and are the first cells to come into contact with the exogenous immune reactive substance. Previous reports have shown that a high-fat diet increases the number of activated Kupffer cells and is associated with the severity of inflammation [50]. In the early stage of liver IR, the release of DAMPs induced by ischemic injury and the binding of TLRs on the surface of Kupffer cells leads to the activation of Kupffer cells [51]. Activated Kupffer cells further enhance the inflammatory response by releasing a large amount of inflammatory cytokines [52].

A study examining that, whether or not HO-1 up-regulation exerts a direct protective effect on active Kupffer cells which aggravate reperfusion injury identified by cobalt protoporphyrin (inducer of HO-1) and zinc protoporphyrin (antagonist of HO-1), respectively. Found that down-regulated the expression of HO-1 by zinc protoporphyrin, the reperfusion injury was aggravated by Kupffer cells activation [53]. The production of inflammatory cytokines and the CD14 expression were decreased in the cobalt protoporphyrin-pretreated group. There was an interesting experiment that has proved the importance of Kupffer cells in IR injury. Kupffer cells and circulating monocytes were ablated by liposomal clodronate in CD11b diphtheria toxin receptor mice, which subsequently suffered liver ischemia. The depletion of Kupffer cells reduces the expression of HO-1 and increases the sensitivity to liver IR injury, but the ablation of circulating monocytes kept the IR injury from becoming more serious. Indicated that Kupffer cells are the main cells expressing HO-1 in the liver and exert anti-inflammatory effects against inflammation-induced oxidative damage, such as IR injury [54].

CO is a product generated by HO-1 degrading heme. It is a signaling molecule and plays a critical role in anti-inflammatory activities, anti-apoptosis activities and vasodilation [55]. The pretreatment of liver donors with CO improves their hepatic IR damage by increasing the number of Kupffer cells and the anti-inflammatory HSP70 pathway expression [56]. Furthermore, HO-1 overexpression in animal liver transplants is mainly achieved through less infiltrating macrophages and inhibiting the expression of inducible nitric oxide synthase [57, 58].

### Activation of neutrophils and lymphocytes

In general, hepatic IR injury is characterized by the recruitment of neutrophils and infiltration into the portal area after ischemia [59]. Acute inflammatory reactions include two consecutive stages: in the Kupffer cell-dominant stage (0–6 h of reperfusion), ROS increase liver injury, and Kupffer cell activation and lymphocyte infiltration induce the secretion of cytokines that further exacerbate the inflammatory response; in the second stage (6–24 h of reperfusion), the neutrophil activation was completely achieved and expressed various types of mediators, including ROS, protease, CXCL-1 and CXCL-2, aggravated liver injury [60]. The retinoic acid receptor-related orphan receptor-γt (RORγt)/IL-17A axis plays an important role in regulating the sub-acute neutrophil-mediated inflammatory responses. The recruitment of lymphocyte and IR injury were found to be diminished in IL-1R1- and IL-17A- knockout mice [61].

Studies on liver injury have identified a neutrophil elastase inhibitor with therapeutic potential that can promote the secretion of high-mobility group box 1 and then reduce the IL-6 expression [62]. In addition, the MMP-9-deficient model of hepatic IR showed an improvement in liver damage that was associated with neutrophil translocation through the hepatic sinusoids [63]. The migration of neutrophils across the endothelial cells and extracellular matrix barriers is a complicated process in hepatic IR injury. CD11b^+^/CD18^+^ neutrophils are very important for the adhesion on the hepatocyte surface and vascular endothelial cells [64]. In addition, CD44 plays an important role in neutrophil infiltration induced by IR injury in mouse liver. Indeed, pretreatment with CD44 antibody reduced the neutrophil infiltration and ameliorated the sinus congestion and hepatocyte necrosis [65]. Although preclinical data have shown good results, a clinical trial of anti-adhesion therapy for IR injury showed no significant improvement [66].

Recently, several investigators have focused on the following three foci concerning the role of neutrophils and lymphocytes in hepatic IR injury: (1) the rapid progress of liver IR injury is not consistent with the time of the T cell response; (2) most CD4^+^ T cells are mainly natural killer T (NKT) cells recruited after liver reperfusion; (3) NKT cells rapidly produce cytokines after stimulation. Shimamura found that the proportion of NKT cells rapidly increases after portal vein clamping-induced IR injury in the liver [67]. The proportion or amount of NKT cells in the total hepatocytes peaked at 10–20 h after reperfusion. The hepatic injury amplitude was reduced by 50% at 6 h after perfusion injury in NKT cell-deficient mice. Interestingly, NKT cells produce interferon-gamma within 2 h after reperfusion [68]. This is due to the fact that NKT depletion in this model reduces biochemical and histological damage, and the adoptive transfer of NKT cells to lymphocyte-deficient mice restores the level of damage to wild-type animals. Experiments have also shown that the aspartate aminotransferase (ALT) in mice treated with synthetic adenosine 2A receptor (A2AR) agonists was reduced by 58%. A2AR agonists can directly reduce the production of IFN-γ by activated NKT cells. All of these results indicated that IR injury and pro-inflammatory cytokine/chemokine transcription levels were significantly reduced following systemic treatment with A2AR agonists [69].

Some investigators have shown that CD4^+^ T cells induce neutrophil recruitment in liver reperfusion injury [68, 70, 71]. However, the mechanism underlying direct tissue injury after liver blood flow recovery is not clear. Using a partial ischemia model, Kuboki et al. found that NKT cells and not natural killer cell (NK cells) play the dominant role in hepatic IR injury through the activation of CD1D-dependent T cell receptor [72]. In contrast, the antibody depletion of NKT cells alone or with NK cells significantly ameliorated liver injury after 8 h of reperfusion. The loss of regulatory T cells has no effect on IR injury. Although the exact mechanism underlying tissue damage is unclear, the release of IFN-γ from the recruited NKT cells can stimulate other proinflammatory cytokines, which may exacerbate liver damage and affect the neutrophil function.

### Apoptosis of hepatocytes

The expression of activated caspase-3 and caspase-9 is high in NAFLD patient specimens. Furthermore, the degree of liver cell apoptosis is notably increased and closely associated with the ponderance of the disease [73]. Hepatocyte apoptosis was found to be positively correlated with liver fibrosis [73]. The present study from Syn WK’s team found that there are three indivisible risk factors associated with apoptosis of NAFLD hepatocytes: dyslipidemia in the liver; cellular stress resulting from changes in oxidation, metabolism and cytokines; and mitochondrial dysfunction [74]. The expression of caspase-3 in plasma and the content of soluble Fas are increased in fatty liver patients. These two biomarkers have a good correlation with liver histopathology [75].

The progress of classical NAFLD is explained by the “two-strike theory”. The first attack is caused by metabolic changes that induce lipid accumulation and steatosis in the liver; the second attack is due to mitochondrial dysfunction caused by metabolism, oxidation, and cytokine secretion [76]. Antioxidants, such as vitamin E and betaine, reduce the level of transaminase via reducing cellular oxidative stress and inhibiting hepatic parenchymal cells apoptosis [77]. Cytokines such as TNF-α, IFN-γ and IL-6 activate the downstream pathway leading to hepatocyte apoptosis [74, 78].

Mitochondrial dysfunction is an extremely important factor influencing the progress of NAFLD hepatocyte apoptosis [77]. It destroys the balance of lipid metabolism in liver cells, mediates oxidative stress and increases the level of ROS. The overproduction of ROS may destroy mitochondrial proteins, phospholipids and even mitochondrial DNA [79, 80]. The mitochondrial DNA depletion of hepatocytes results in a reduction in the expression of mitochondrial DNA-encoded polypeptides and increased mitochondrial dysfunction [81].

Apoptosis and necrosis are common reactions in the liver when suffering injuries induced by ischemia, radiation and poisonous substance [30–32]. The liver, especially the hepatic parenchymal cells, is sensitive to damage caused by IR injury [33, 34]. In IR injury, the occurrence of oxidative damage in the enzyme complexes and the decrease in the anti-apoptotic protein level cause apoptosis of hepatic parenchymal cells [35]. The inhibition of the caspase family was found to notably attenuate hepatic injury induced by IR, indicating that apoptosis plays a key role in IR damage [36]. Hepatocyte apoptosis leads to elevated AST and ALT levels and further reduction of the liver function [37].

A liver IR injury rodent model was generated by 1-h portal vein occlusion and 24 h of reperfusion. The hepatocyte apoptosis was markedly increased in the liver IR injury group compared with the sham operation group and became more aggravated over time (55-fold increase after 4 h of reperfusion and 200-fold increase after 24 h of reperfusion) [82]. The interaction between various types of hepatocytes, like parenchymal cells, Kupffer cells and neutrophils, promotes hepatic IR injury [53].

Liver IR damage induces hepatocyte apoptosis and produces a series of changes, including oxygen-free radical production, calcium overload and changes in the permeability of mitochondrial membranes and the expression of cytokines and apoptotic genes [45, 83, 84]. Ca^2+^ overload, anaerobic metabolism, acidosis and oxidative stress trigger hepatocyte apoptosis during hepatic IR injury. Intracellular Ca^2+^ overload activates Ca^2+^-dependent enzymes, eventually leading to cell apoptosis [85]. Oxidative stress causes mitochondrial dysfunction and lipid peroxidation and induces apoptosis by stimulating the production of active molecules, like ROS [86].

Molecular hydrogen, a new type of antioxidant, ameliorated hepatocyte apoptosis in hepatic IR injury by inhibiting the level of oxygen radicals [87]. The process of aerobic and anaerobic metabolism inhibits redox reaction in hepatocytes, resulting in the depletion of intracellular ATP. This shortage of energy leads to mitochondrial damage and microcirculation failure. Enhanced anaerobic glycolysis reduces the intracellular pH and ultimately leads to apoptosis associated with acidosis [88]. A number of cytokines are involved in the regulation of apoptosis, including Bax (pro-apoptotic factor) and Bcl-2 (anti-apoptotic factor) [89]. In steatosis hepatocytes suffering from hypoxia/reoxygenation injury, the Bax expression was found to be significantly up-regulated, while the expression of Bcl-2 was dramatically down-regulated [90].

The overexpression of HO-1 exerts strong cytoprotective functions in many liver IR injury models. The expression of HO-1 in normal or fatty liver transplantation or after transplantation can be increased by drugs or gene engineering, which maintains the tissue structure and organ function and prolongs the survival time of the graft [57]. The overexpression of HO-1 reduces the hepatic apoptotic IR damage by reducing the C/EBP homologous protein levels and expression of NF-κB mediated genes, such as MCP-1 and IL-6, while increasing the IκBa expression (repressor of NF-κB) [91]. Adenovirus-mediated HO-1 overexpression was reported to increase the expression of anti-apoptotic molecule BAG-1 and reduce the number of apoptotic cells in mice [92]. Interestingly, the expression of Bcl-2 and BAG-1 (anti-apoptotic genes) was increased in grafts with a good liver function, while the Caspase-3 expression was significantly decreased in such grafts. Obviously, the cytoprotective effect of HO-1 is directly related to the increase in the expression of anti-apoptotic genes [93]. CO, the degradation product of HO-1 can reduce the apoptosis induced by IR damage through synergistic action of miR-34 A/SIRT1 signaling pathway [94].

## Novel strategies for preventing fatty liver IR injury using an HO-1 inducer

### Aminolevulinic acid (5-ALA) and sodium ferrous citrate (SFC)

5-ALA is an important intermediate product of heme synthesis and is the basic substance of aerobic energy metabolism. It acts as a precursor of photosensitizers for a photodynamic diagnosis (PDD) and photodynamic therapy (PDT) to identify and kill tumor cells [95]. In the human body, 5-ALA is synthesized to protoporphyrin IX (PpIX) in the mitochondria, and divalent iron ion is inserted into PpIX to form heme. Hayashi et al. showed that the usage of SFC as a form of iron enhanced the specific effect of 5-ALA in tumor PDT [96].

Several studies found that the coalition of 5-ALA and SFC mediated the overexpression of HO-1 [97, 98]. Pretreatment with the coalition of 5-ALA and SFC resulted in the increased expression of HO-1 in mouse kidney, and CO produced by the degradation activity of HO-1 showed a protective effect against IR damage in kidney [97]. Previous findings obtained by the authors have shown that application of the precursor of heme anabolism (5-ALA) along with the byproduct of heme catabolism (ferrous iron) in the macrophage cell line RAW264.7 up-regulated the HO-1 gene expression to produce an anti-inflammation effect [99]. Our laboratory’s data indicated that the treatment of coalition of 5-ALA and SFC ameliorated the IR damage in steatosis livers. The protective effects were mediated by the high concentration of CO, and ROS were reduced by inhibiting the TNFα/NF-κB pathway [99].

### Astaxanthin (ASTX)

Astaxanthin (ASTX) is a xanthophyll carotenoid, that can be produced by a variety of seaweed and microorganisms [100]. ASTX has many antioxidant properties because it has a unique and remarkable molecular structure of hydroxyl and keto moieties on each ionone ring [101]. The antioxidant properties of ASTX have been previously observed in the plasma, eye and liver of rats fed microalgae biomass, which contained ASTX, dispersed in olive oil. Furthermore, the levels of antioxidant enzymes were up-regulated when ASTX was administered to ethanol-induced gastric ulcer rats [102]. In addition, an in vitro study showed the beneficial effect of ASTX in tubular epithelial cells stimulated by high glucose, which induced inflammation and oxidative stress [103].

Some reports have shown that ASTX dissolved in olive oil had no negative effects and in fact exerted protective effects against oxidative stress [104]. Recently, Ni et al. reported that ASTX was more useful for preventing and treating steatotic liver than vitamin E in mice [105]. Furthermore, several reports have shown that ASTX attenuates hepatic [106], retinal [107], and renal [108] IR injury in rodent models. Our previous study demonstrated the protective effect of ASTX in steatotic liver affected by IR injury. The histopathological features of IR injury, including necrosis, apoptosis infiltration of macrophages, lipid peroxidation products, liver enzyme leakage and inflammatory cytokine expression, were corrected by ASTX. The inhibition of ROS production and inflammatory cytokine expression, induction of HO-1/Nrf-2 in Kupffer cells and inactivation of MAPK may be involved in the inhibitory mechanism of ASTX against IR injury in the steatotic liver. The decrease in the expression of cleaved caspase-3/9 and Bax and the up-regulation of Bcl-2 in hepatocytes may also play a role [90].

### Molecular hydrogen

Molecular hydrogen has a novel medical application. Similarly, nitric oxide and CO, which exert cytoprotective effects against cellular stress, have also drawn attention [109]. Molecular hydrogen exerts cytoprotective effects in the nervous, cardiovascular and digestive systems [110–112]. According to our unpublished data, hepatic protection by molecular hydrogen was observed in a mouse model of fatty liver IR, and hypoxia/reoxygenation-induced damage was observed in fatty hepatocytes in an in vitro model. Hydrogen saline exerted marked hepatic protection by preventing hepatocyte death and inhibiting macrophage recruitment compared with mice treated with normal saline. Furthermore, its application inhibited hypoxia/reoxygenation-induced damage in fatty hepatocytes. During ischemia/hypoxia and subsequent reperfusion/reoxygenation, a large number of harmful substances, such as pro-inflammatory cytokines, which produced by activated Kupffer cells then resulting in hepatocyte apoptosis.

Recently, some researchers have found that molecular hydrogen exerts potent pharmacological effects by reducing oxidative stress, inflammation and apoptosis [113, 114]. Molecular hydrogen has emerged in the form of hydrogen-rich water or inhaled hydrogen gas and is recommended as an effective treatment for cardiac arrest in the clinical setting. There are many ways to administer molecular hydrogen, with oral treatment being the most convenient, although some hydrogen gas will escape into the stomach via this route. The administration of hydrogen saline to the target tissue allows for more efficient molecular hydrogen delivery [115]. A recent report derived from rat models has highlighted the novel, promising therapeutic application of imaging-guided hydrogen bubble delivery to prevent myocardial IR injury [116].

Growing evidence indicates that the protective effect of molecular hydrogen is not only interrelated with the elimination of oxygen-free radicals but also associated with various intercellular signals [117, 118]. Kawamura et al. reported that hydrogen gas ameliorated lung injury by promoting the expression of Nrf-2, which contributes to HO-1 expression [119]. Cai et al. [120] revealed that molecular hydrogen therapy relived TNF-α-induced rat osteoblast inflammatory injury via the down-regulation of the NF-κB pathway. Furthermore, there is also evidence that molecular hydrogen reduces lung injury induced by transplantation by increasing the expression of HO-1 [119]. In our research, hydrogen saline exposure was found to significantly increase the expression of HO-1 according to in vivo data. The protective mechanism underlying hypoxia/reoxygenation-mediated hepatic injury was clarified in an in vitro study. Our results demonstrated that HO-1 positively regulates the expression of Sirt1, while HO-1 and Sirt1 attenuate the Kupffer cell activation. Sirt1 inhibits the activity of the p53 pathway by directly inducing the production of the apoptosis regulator Bcl-2 and inhibiting the transcription of Bax and the activation of cleaved caspase-3.

## Conclusion

**Fig. 1:**
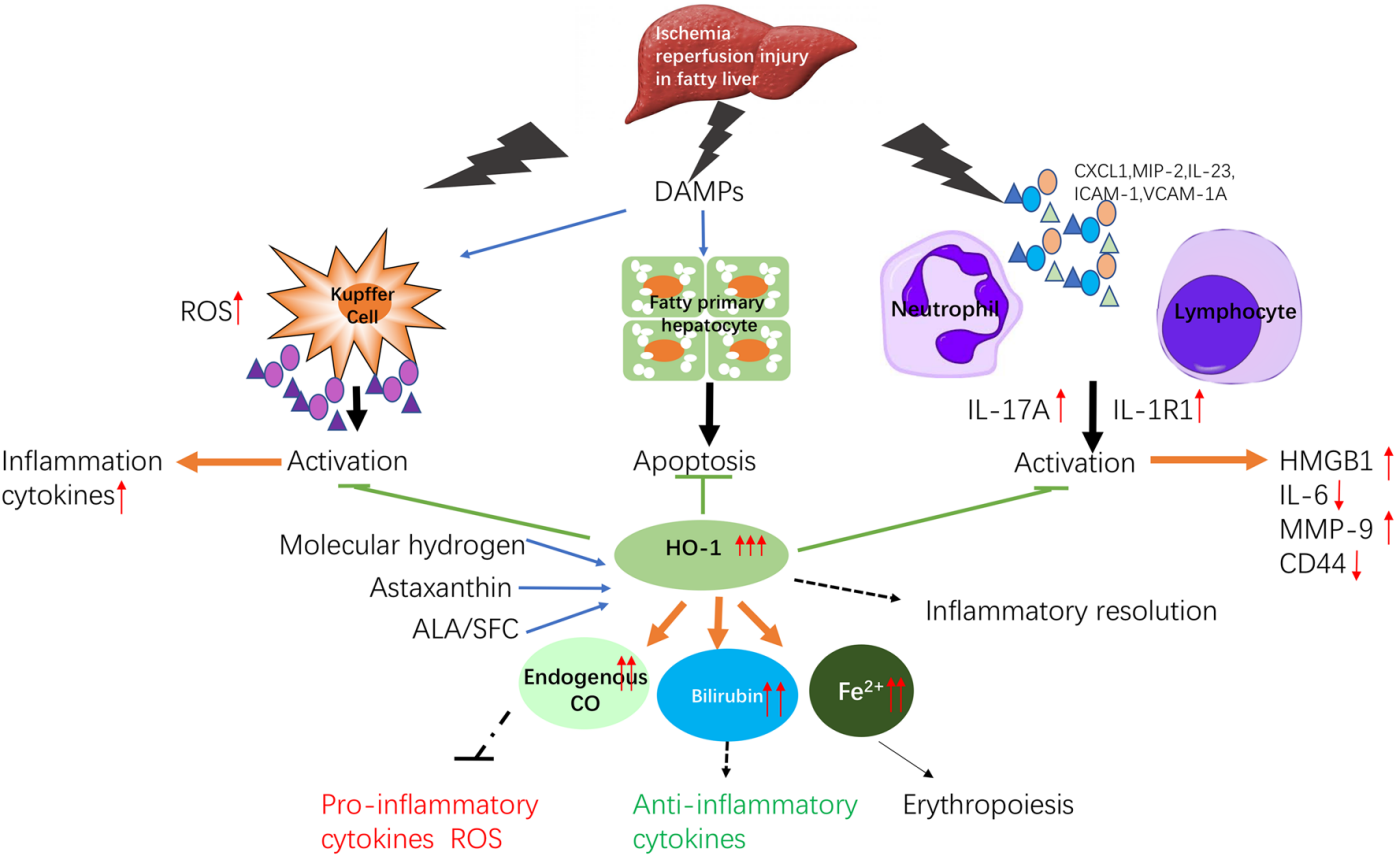
Scheme of the mechanism of IR injury liver in fatty liver and protection by HO-1

Figure 1 summarized the mechanism of IR injury in fatty liver and HO-1 protection of it, which is described in this review. Liver IR damage, especially the IR injury of fatty liver, is a complicated, multi-factor pathophysiological process. However, despite its complexity, the HO-1 signal pathway may play a critical role in a variety of pathophysiological conditions owing to it’s antioxidant, anti-inflammatory, anti-apoptotic properties. Therefore, the pharmacological regulation of the HO-1 signal pathway may be an effective clinical strategy for reducing fatty liver IR damage.

HO-1 amplifies a variety of cellular protective mechanisms against various intracellular stresses. The antioxidant effect of HO-1 is achieved through the degradation of heme into CO, biliverdin/bilirubin and Fe^2+^. CO, the final product of HO-1 degradation, exerts a significant anti-inflammatory effect by reducing Kupffer cell activation and exerts an anti-apoptotic effect in steatosis hepatocytes through the activation of p38 MAPK. CO also regulates the vascular tone, resulting in decreased platelet aggregation. The lack of clinical studies on the impact of liver IR injury on NAFLD patient outcomes has left a number of questions unanswered, and we will need to conduct a large number of prospective trials to clarify several points. Based on the extensive experimental evidence, liver surgeons should be careful to avoid liver IR damage in NAFLD patients and actively seek new ways to improve their injuries.

## Abbreviations

IR: Ischemia–reperfusion
NAFLD: Non-alcoholic fatty liver disease
HO-1: Heme oxygenase-1
CO: Carbon monoxide
ROS: Reactive oxygen species
TNF-α: Tumor necrosis factorα
IL: Interleukin
NF-κB: Nuclear factor kappa B
HMGB1: High mobility group box-1
VCAM-1: Vascular cell adhesion protein 1
DAMPs: Damage-associated molecular patterns
TLR: Toll-like receptor
5-ALA: 5-Aminolevulinic acid
ASTX: Astaxanthin
